# Death Rate of the Rich and Poor

**Published:** 1881-11

**Authors:** 


### POPULAR SCIENCE DEPARTMENT.

### DEATH RATE OF THE RICH AND POOR.

All the sanitary improvements have not reduced the death rate of London, for during the past few years the rate was as follows: In 1856, 22.2 per 1,000; 1876, 22.3; 1877, 23 per 1,000. In all England the rate had remained identically the same for three decades, namely, 22.35 per 1,000. The point endeavored to elucidate was, that the great cause of this non-improvement resided in the mass of indigence which now, as ever, was instrumental in producing a large crop of premature deaths in all densely populated States. Thus, it has been observed in France that person between the ages of 40 and 45 die,if in easy circumstances, in the proportion of 8.3 per 1,000; while in poor, they died at the rate of 18.7 per 1,000; that is,the mortality between these ages was twice and a half as large among the poor as it was among the wealthy. It was found too, that in Paris, between the years 1817 and 1836, 1 inhabitant in ever 15 died in the twelfth arrondissment, which is peopled in great part by the poor; while in the second arrondissment, inhabited by the wealthier classes, the deaths for the same period were only one in every 65. M. Garnier,of Paris, in 1857, speaking of the mean life in a large English manufacturing city, had found that it was only 17 years in the quarters inhabited by the poor, against 42 among the higher classes. Villerme calculated that the probable life of the infant of a weaver at Mulhouse was as low as one year and six months; while that of the baby of the proprietor of the factory was 26 years. Dr. Drysdale cited from a pamphlet written in 1877, upon the dwellings of the wages-receiving classes in Paris, some further suggestive figures, from which it appeared that a death rate which was the mean of the whole population is always misleading. Thus, in part of a sub-district in London comprising houses in good condition, the death rate did not exceed 11.3 in every 1,000; while there were adjacent dwellings in the same sub-district in which the death rate had risen to 38 per 1,000; and it was now reported that there were particular districts in London where the death rate was 50 per 1,000. On the other hand, the average death rate of the whole population was only 24 per 1,000 in 1843, and had scarcely deviated from that figure since. If such statistics were insufficient, he would refer to the researches of Ansell, who collected the statistics of 48,044 of the opulent classes in England, including professional men, the nobility and gentry. It appears from Ansell's tables that among these classes the death ratio was only 80.45 per 1,000 for children under a year old, while for all classes taken together it was 150. Dr. Little found the ratio in Berlin, a city of extreme poverty among the working classes, to be occasionally as high as 500 per 1,000. In conclusion Dr. Drysdale referred to the statistics of New Zealand as a remarkable confirmation of Ansell's tables. In New Zealand, of late years, the wages of laborers had been very high, and the profits of capital large, with meat only 3d. per pound, so that the laborer was able to secure plenty of food without undue anxiety. The result was a death rate of only 12.5 per 1,000—a fact mainly due to the absence of an indigent and badly paid class. In England and Wales, with the same death rate, some 230,000 lives would be saved every year.

